# Understanding the mental health and wellbeing needs of police officers and staff in Scotland

**DOI:** 10.1080/15614263.2020.1772782

**Published:** 2020-06-09

**Authors:** Evangelia Demou, Hannah Hale, Kate Hunt

**Affiliations:** a MRC/CSO Social and Public Health Sciences Unit, Institute of Health and Wellbeing, University of Glasgow, Glasgow, UK; b Institute for Social Marketing, University of Stirling, Stirling, UK

**Keywords:** Police, mental health, workplace intervention, stress, PTSD, stigma, occupational health

## Abstract

Police work can be stressful and demanding and can impact on employee wellbeing. This study aimed to understand mental health (MH) issues and risk factors for poor MH in officers and staff of the Police Service of Scotland (PSoS); and gather their ideas of workplace wellbeing interventions that are suitable for this workforce. Face-to-face/telephone interviews were conducted with 30 Superintendents and eight stakeholders, recruited throughout PSoS. Interview topics included: MH issues; health/health behaviours; employment; and potentially beneficial workplace interventions. A thematic analysis approach was adopted. High levels of occupational stress and anxiety, currently or in the past, were reported, as were experiences of PTSD, anxiety and depression. The main stressors reported were working hours, workload, culture, leadership and organisational change. Officers and staff recognised progress towards promoting and managing MH in the service but identified interventions, including training, counselling, and environmental workplace changes as needed to address mental health issues within police cultures.

## Introduction

Police officers’ roles and responsibilities place them in challenging and stressful situations that can significantly impact on their mental wellbeing and possibly even performance (Garbarino et al., 2013). Evidence suggests that the ways in which mental health (MH) challenges manifest or are dealt with in police forces differ from other occupational groups (Garbarino et al., 2013; He et al., 2002; Kapade-Nikam & Shaikh, 2014; Kumarasamy et al., 2016; McCarty et al., 2007; Tewksbury & Copenhaver, 2016). Research on the mental health (MH) problems police face, reports a range of issues, including depression (Garbarino et al., 2013), stress (Goodman, 1990; Kumarasamy et al., 2016), PTSD (Violanti et al., 2007), somatization (He et al., 2002), burnout (Garbarino et al., 2013), anxiety (Garbarino et al., 2013), and family problems (Kapade-Nikam & Shaikh, 2014). A range of risk factors contribute to the stress and distress experienced by police officers; these are often divided into two categories: operational and organisational stressors (Alexander et al., 1993; Evans & Coman, 1993; Purba & Demou, 2019). Organizational stressors have been identified as more likely to cause adverse psychological distress than operational stressors (Brown & Campbell, 1990; Brown et al., 1999; Kop & Euwema, 2001; Tyagi & Dhar, 2014). Our recent systematic review demonstrated strong evidence of associations between organisational stressors and occupational stress, psychiatric symptoms/psychological distress, emotional exhaustion and personal accomplishment (Purba & Demou, 2019). The main work-related stressors impacting on the mental health of this key group of employees were long working hours and heavy workload, police culture and organisational change (Purba & Demou, 2019). In another study on police stress interventions, Amaranto et al. observed that prominent sources of direct stress included: ‘(a) being “second-guessed” in field work, (b) punishment for “minor” infractions, (c) lack of rewards for jobs well done, (d) fear of being “de-gunned” (official removal of an officer’s service revolver as well as any personal weapons) for stress or personal problems, and (e) low morale’ (Amaranto et al., 2003). Acute stress has been demonstrated to cause PTSD among other outcomes, contributing to poor health even after officers retire (Violanti et al., 2007). High-job demands placed on this occupational group are often associated with burnout, resulting in knock-on effects on sickness absence levels, greater use of force and poor interactions with the public, health issues, strained relationships and lower quality of work (Manzoni & Eisner, 2006; Richardsen & Burke, 2007). In addition, organisational structures and cultures influence beliefs, attitudes, identities, and cognitions and can affect employees’ ability to undertake their roles or their response to stressors (Garbarino et al., 2013; He et al., 2002; Kapade-Nikam & Shaikh, 2014).

Police officers can be especially vulnerable to poor MH if they do not have support systems (family, friends, peers, trust from colleagues and supervisors), or if they lack personal qualities that enable them to cope (Loriol, 2016). Organisational cultures and fear of stigmatisation are other critical factors affecting officers’ MH (Bell & Eski, 2016; Garbarino et al., 2013). Gabarino et al. suggested that ‘[i]nvestigating stress in police officers is particularly difficult because they are afraid of being identified as individuals who have been compromised by stress’ (Garbarino et al., 2013). Officers are encouraged to display physical and emotional courage and a lack of emotional control can be deemed a weakness (Garbarino et al., 2013), potentially leading police officers to suppress their emotions (Bell & Eski, 2016; Bonifacio, 1991). The current culture in police forces and amongst other emergency personnel, can make it difficult for individuals to disclose and discuss mental health issues and this ultimately poses a barrier to receiving support (Bell & Eski, 2016; Berg et al., 2006). Disclosing mental health problems has been described as ‘career destroying’(Bell & Eski, 2016). This is further supported by Bell and Esky who suggested that the inherent cynicism, lack of empathy, and macho culture that have been associated with policing cultures, deter discussions around mental health issues and access to support (Bell & Eski, 2016). The implications of this stigma can be far-reaching, leaving those experiencing MH issues feeling isolated and marginalized (Bell & Eski, 2016). Power and gender dynamics also contribute to constructions of police culture (Morabito et al., 2011). McCarty (2013) suggests that in broad terms, masculine culture has pervaded law enforcement organisations, resulting in an environment in which female officers may feel uncomfortable.

Work-related mental health problems have several socioeconomic consequences due to factors such as sickness absence, presenteeism, leaveism, loss of productivity and ill-health retirement. In addition to economic costs, personal costs to the individual and their families include lower self-esteem, somatization and a negative impact on family and social relationships. The workplace is one of the most important settings for mental health promotion and behaviour change. Workplace interventions aimed at mental health protection are implemented mainly at the *organisational level*, targeting working conditions and policies, or at the *individual level* through programmes on stress management and skills training to provide employees with the tools and resources to cope with the impact of work-related problems.

Numerous studies examine interventions developed or tailored for the police, as well as generic workplace interventions implemented within police workforces (Acquadro Maran et al., 2018; Arokoski et al., 2002; Backman et al., 1997; Doctor et al., 1994; Carlier et al., 2000; Carlier et al., 1998; MacMillan et al., 2017; Norris et al., 1990; Patterson et al., 2014; Penalba et al., 2008; Richmond et al., 1999; Shipley & Baranski, 2002; Tolin & Foa, 1999; Verbeek et al., 2018; Wilson et al., 2001). Most of these interventions report varying success and study limitations, such small sample sizes. A systematic review examining manualised psychosocial interventions to prevent psychological disorders in law enforcement included: cognitive-behavioural-theory interventions; supportive therapies; psychodynamic and physical-activity therapies and non-pharmacological strategies (Penalba et al., 2008). Two studies have examined the impact of debriefing as an intervention following critical incidences (Carlier et al., 2000; Carlier et al., 1998). Both studies showed no difference in psychological morbidity or post-traumatic stress symptomology following debriefing. However, at follow-up subjects in the intervention group (debriefed) exhibited *more* PTSD symptomatology than non-debriefed subjects (Carlier et al., 2000; Carlier et al., 1998). While the outcomes did not show effectiveness for this type of intervention, there was a high level of satisfaction from the intervention subjects (Carlier et al., 2000; Carlier et al., 1998). Group counselling demonstrated no significant changes in general health or sickness absence, although the sessions were valued by police officers (Doctor et al., 1994). Conversely, an intervention using training in psychological and technical techniques to reduce anxiety and enhance operational performance in US police officers, demonstrated positive findings that were sustained two years post-implementation (Arnetz et al., 2013). Another study found that physical activity and wellness courses resulted in decreased perceived stress and increased wellbeing (Acquadro Maran et al., 2018).

Many recent studies highlight the range of mental health issues experienced by people working in police organisations across the world and evidence suggests that the ways in which mental health challenges manifest in police workforces differ from other organisations. However, to date, little research has been conducted in the UK on MH issues in the police forces (Bell & Eski, 2016; Houdmont et al., 2018; Johnson et al., 2005). Recent Police Service of Scotland (PSoS) staff surveys have identified that MH issues, such as anxiety and depression, are a concern, as are a number of organisational risk factors which impact on MH, wellbeing and family life (Association of Scottish Police Superintendents, 2015). Our study aim was to conduct an exploratory study within the PSoS to: understand the MH issues officers and staff face and the perceived risk factors for poor MH; and assess what policies, practices and interventions police officers and staff think are appropriate and can be effective in their organisation. Understanding the mental health issues and their key risk factors for police forces has immense potential for the wider study of the long-term health of a key front-line service.

## Methods

The setting of our research project is the Scottish Police Authority/The Police Service of Scotland (PSoS). This is an organisation of over 23,000 employees (Police Officers, staff and Special Constables). In-depth semi-structured interviews were conducted with PSoS employees and stakeholders (line managers, management/human resource personnel, trade union representatives, others involved in workforce wellbeing), either face-to-face or by telephone. Our PSoS employee target population was the Superintendent rank who, as a group: are exposed to many traumatic incidents; have experience of the demands and risk factors in the lower police ranks, experience high levels of demands; and are likely to line-manage more junior staff. Currently there are approximately 160 Superintendents in the PSoS. The majority are between 46 and 50 years of age and approximately 70% have been in this rank for 1–5 years. Therefore, they can both reflect on their own experiences and opinions as officers and provide insights into work-related issues for lower ranks of the workforce. We aimed to conduct up to 40 c.60-minute interviews with Superintendents and 10 ‘stakeholder’ interviews. We sought interviews with people with personal experience of MH issues themselves or of managing people with MH issues at work. Interviews with Superintendents covered topics including: MH-related issues (both work and non-work related); general health, wellbeing and health behaviours; employment; opinions and ideas on what workplace interventions for MH issues work/do not work. Interviews with stakeholders explored similar issues, and additionally: how MH-related issues differ for officers and staff; mechanisms in place to identify and manage MH conditions; perceptions on whether current support was effective; and perceived facilitators and barriers to offering new workplace MH interventions.

Participants were recruited from locations throughout the PSoS. The study was advertised using posters and a brief notice distributed via the PSoS. The researchers offered to answer any questions before participants gave permission to take part and were asked to sign a consent form or provide recorded verbal consent over the telephone. Interviews were audio-recorded using an encrypted dictaphone with participant consent. Two members of the research team conducted the interviews, one undertaking interviews only with the Superintendents and the other with the stakeholders.

Two researchers (HH and ED) independently read a sample of anonymised transcripts to develop an analytical framework and identify key themes. Transcripts were then read repeatedly and coded for analysis using QSR NVivo12. A thematic analysis approach was adopted (Braun & Clarke, 2006); data were coded and indexed into six primary categories: perceptions of MH, personal experiences of MH, MH stressors, police cultures, MH policies/practices, and MH interventions. All participants gave their permission for anonymised quotes to be used. Quotations attributed to Superintendents are denoted by P and stakeholders by S. The numbers used only differentiate participants and are randomly allocated with no links to the order in which interviews took place, job title, gender, etc. We use ‘s/he’ and ‘him’/her’ throughout as an additional measure to protect against (presumed) deductive disclosure of participants’ identities.

## Results

Thirty Superintendents were interviewed (n = 20 men, n = 10 women). Twenty-five worked mainly in urban environments, three in rural areas and two had worked in both contexts. Participants had 18 to 29 years of service with the police and the majority had 5 years or less years of service before their planned retirement. The eight stakeholders interviewed (n = 6 women, n = 2 men) had a wide range of experience within the PSoS and covered strategic roles, including in policy development and implementation, wellbeing remit and management.

When asked about their perceptions of good and poor MH and personal experiences of MH issues, participants felt that, although there was still room for improvement, police officers’ understandings of MH issues had improved considerably. Their perceptions of the concept of MH varied: some framed MH as the (in)ability to cope with stress and several referred to ‘struggling’, ‘stress’, ‘mental ill-health’, and ‘crisis-situations’ (see officer participant quotes below). The definition of MH was also linked with ‘happiness’.

***P12:** “ … So fundamentally if policing is a people business, each and every one of the people who are in it need to have good mental health to enable them to function and deliver that service. It is a fundamental core responsibility for the leadership to equip, support, encourage every single person to be the best person they can be, to navigate their way through their daily life, and the ten, twelve, fourteen hours they do in the job. To do anything other than that is a failure of leadership.”*

***P1:** ‘ … your mental ability to actually cope with what’s going on every day in your life.’*

***P28:** “ … MH, the first thing that comes into my head is when somebody’s struggling, … It’s probably as simple as I can put it.”*

***P30:** “For me probably, the first … when somebody says … asks me about my mental health, I immediately think of my stress levels, that’s the first thing I think on and probably my wellbeing, how do I feel, kind of almost a happiness factor effectively … ”*

***P22:** “ …. So my, kind of upbringing in the police service and just general society … society’s reference to mental health immediately conjures up pictures and a perception of individuals and a perception of men … mental ill health.”*

As seen in their following quotes, stakeholder perceptions of what MH meant to them appeared to centre more around overall wellbeing and feelings of happiness, or to an officer’s personal response to traumatic operational situations:

***S8:** “ … for the workforce in general, … good mental health … is their wellbeing, it’s about people being happy and content at their work, about not suffering from anxiety or … depression, or … other manifestations.”*

***S7:** “Well I think it means a number of things, but I think, er, in the first instance it’s … it … it means something about, um, the health and wellbeing of an officer, both mentally, emotionally and probably to a lesser extent physically. Um, and then probably for me is about an officer’s ability to … or a member of police staff’s ability to perform their duties, um, is the first thing and the second thing is it’s something about […] … how they respond and react to situations that they have to deal with, so instances … obviously, they were very, very traumatic. So it’s about giving consideration to the impact of the, kind of, day-to-day role of being a police officer.”*

All participants appeared open and candid when asked about their experiences of MH. Many expressed concerns about the MH of the PSoS workforce; and highlighted challenges relating to depression, stress, anxiety and PTSD, as seen in the quotes describing lived experiences of mental health issues below from officers and stakeholders.

***P21:** “Yeah. Because, you know, most of the sickness is related to stress, it’s got to be. So, it makes … all stress related stuff, you know, not coping, bursting into tears, you know, that kind of stuff, it’s all quite prevalent within the service. So, we need to get better, but I think it’s pretty good, to be honest with you. I think it can get better, at least”.*

***S4:** “ … for myself, my own diagnosis, […], I have generalised anxiety disorder, so I worry about everything and nothing in particular. […] and when I was diagnosed, erm, I was also diagnosed with depression, severe depression. […] I was quite happy to accept the anxiety because that made sense to me. And, and I did feel anxious but I certainly rebelled against the depression bit”.*

***P12:** “The implications of not feeling value and not being able to fulfil one’s potential or one’s purpose in life, working life, undermines your … potentially undermines your feeling of self-worth and your identity if you allow it to. If you can’t process that and understand that, and have the mental capacity to not allow it to drive you to depression … ”*

Depression was linked to work organisation, exposure to critical incidents, and to a lack of ‘feeling valued’. A large majority of officers experienced stress and conveyed both personal and work challenges and associated impacts on job performance and home life. Concurrently, many remarked that they had not informed anyone within the police force of this. Anxiety was a prominent concern and issue for a significant number of participating officers and staff and its prevalence was thought to be vastly underestimated, as expressed by P15 below:

***P15 (Lived-experience of mental health issues):** “So I’ve had this experience where I’ve now discovered I’ve got some kind of mental health anxiety which is leading to … I’m doing something about it. […] The only time I’ve actually ever had to go and get diagnosed with it was [when] I had to get them to write it down because it had to suit me to then to go and get help about it so … Erm, but I recognise in everybody else in almost probably sixty or seventy per cent of the people that I talk to – I actually suspect it might even be more than that – is that they have the same symptoms that I have”.*

While some officers explicitly stated they had suffered from PTSD symptoms, others recounted experiences or described symptomatology typical of PTSD but these were often normalised and accepted as simply ‘part of their job’. Relaying memories of past traumatic operational incidents still evoked emotional responses, which at times surprised participants, as seen in the participant quotes below.

***P21:** … there’s things can trigger it, erm, you know, smells especially, you know. Erm, it’s just, I don’t know what it is.[…] I think you’ve just got to, I think you’ve got to just calm yourself down, and use, kind of, breathing, to be honest with you. […] Because your heart rate goes up, and you start sweating, and, erm, that’s my signs. My signs are, heart rate, sweating, and then, erm, you know, you can’t quite … so somebody’s talking to you, but you’re not hearing a word they’re saying, you know, that kind of thing. Because your mind’s away. It can happen at any time, because of the things that I’ve seen are beyond belief. Actually, beyond belief, you know, things like no normal person would ever imagine the things that we do, as a CID officer. […] I mean, you’re dealing with death, every day, but death, violent death. And it’s a really hard thing to cope with …*

***P22:** “Um, but there was one particular incident that I dealt with, um, years and years ago and it was a fatal collision involving a little girl. Um, and I … I won’t go in to details because it was especially gruesome, but I dealt with it, recovered her, um, dealt with it and […] it was fine. And like everything else, you move on […] and the police service dealing with these things in the way that we do, we tell jokes, um we go for a curry, we go for a pint um, and we just get on with it. And I was fine. […] years later […] my daughter was born and, um, that was fine. She’s a baby. That’s fine. And then she starts to develop into a little girl and I’ll never forget the day when I … I went to pick her up or place her in her bed and, um, I describe to people … and this is … this is probably the primary reason that I said I would take part in this … um, it … I felt as if I’d been hit over the back of the head with a cricket bat … and I just saw this little girl again … And after that, I … […] really struggled and I had a period off work and I … I did suffer from depression and anxiety and a […] lack of confidence, um … in my ability as a […] supervisor and even as a [spouse] and a […][parent] … to look after my family. But also if I can’t hold this together, how can I look after my shift? How can I look after my men and women at work if I’m struggling to hold things together?”*

Operational experiences where fellow officers were directly affected or even killed, were especially challenging. Stakeholders thought that experiences of PTSD and PTSD symptomatology among officers were very common. Participants relayed how their job and their MH impacted on family life and raised concerns around how difficult it is to achieve and maintain a good work-life balance, as participant P28 states below. We explore this in more detail in a follow-up paper.

***P28 (work-life balance):** “ It was rubbish then. I would get home and kiss my [child] good night, and I’m up before [child] goes to work, I’m still up before [child] gets up, so I was away and I’d get home sometimes to read [child] a story, and so work life balance was … I mean, it was non-existent, then I was unhealthy.”*

The operational and organisational stressors considered most common among officers and staff, were: traumatic operational incidents; the fast reaction times needed when handling challenging incidents and how this can be taken for granted; job role (including rank, lack of role clarity and appraisal, ineffective handovers); workload (including staffing, resources, public perceptions); working hours (including long-working hours, on-call, short notice shift changes); police cultures (including stigma, help-seeking behaviours, confidentiality, ‘masculine’ culture, ‘cupboarding’, bullying); and organisational structures and change (including increased scrutiny, performance, media pressures, leadership, management, lack of training), with emphasis given to the merging of different forces to create Police Scotland in 2013. Stakeholders considered operational stressors to be most challenging for officers, while officers maintained that organisational stressors presented the greater challenge to their MH. Some of these risks to their mental health were described in the following quotes:

***P26 (Traumatic incidents):** “ … when I, erm, came back to operational policing as a Sergeant, I was a [parent] and I knew that I was a completely different officer from before it and the defining moment of that was going to a road accident […] and the wee boy had been playing in the … the [name of field] … He’d been playing football with his big brothers and stuff and he had kicked the ball over the fence and it had went … […] he ran out straight into the middle of the traffic and got ran over. Wasn’t dead, thankfully […] and, erm, his mum […] she arrived and at … and at that defining moment, seeing another [parent] with a … a wee boy, I had a wee boy, and he’d been knocked down and she was just frantic with panic and worry and concern. I mean, I’m getting goosebumps even thinking about [it and] I remember think[ing] … and I knew exactly how she was feeling because I was a [parent] and I also knew exactly, when I reflected on it later that night, I also knew exactly how I didn’t feel at those types of incidents which I’d went to plenty of times when I wasn’t a [parent] and I think, oh my God, I dealt … I dealt with that completely differently.”*

***P21: (Organisational versus operational risk factors):** “Organisational. Oh, it’s way worse. […] Operational stress is easy, because that’s, we’re good at that. […] I can deal with anything operationally, every day. Give me, the more they give me, the happier I am. I love dealing with stuff, and sorting things out, and solving cases. So that’s, that’s the bread and butter, that’s the easy stuff. The pressure, though, is ridiculous, and it’s ridiculous organisational bureaucracy, and, and pointless exercises that take place, for some statistical report. That’s really frustrating for a police officer. And that’s massively stressful.”*

***P1 (Working hours, workload and work demand):** “I … I feel that I’m going to burn out my own staff that I’ve got … if I don’t fix it. So that’s probably where my challenges are. Um, about trying to get a bit of traction and understanding about how much work they’re dealing with and how few people are doing it. […] The expectation is huge of what we expect them to do every day … and they all do it really well … but they do … you hear the rumblings in the background but even just me physically looking at how few people there are and how much work they get done, it’s just almost impossible … It’s incredible. So I need to try and fix that, so that’s probably … and that alone in the, sort of, financial climate we’re in … ”*

Police officers know that support for their wellbeing is available but described how the stigma associated with MH issues in the police prevents them from seeking help and being open about their difficulties, as described in their quotes below. Participants acknowledged that MH stigma was still present but not as strong as previously.

***S4:** “Yeah, erm, I, I think the cynicism is one, erm, the stigma that would be attached if someone was to find out that someone had contacted, erm … There is also a bit of the culture because it’s male dominated that, erm,[other] people are worse off than me and they are not getting help so I don’t need it, or they’ll look to other methods to medicate rather than talk to someone. Erm, so that can be a barrier to intervention. The barrier is also confidentiality because people don’t believe it’s a confidential service and, erm, if someone has had a bad experience of maybe accessing the service … we had to really manage expectations at one point.”*

***P17:** “I think there is a stigma. Is the stigma as bad as what it was previously? No, but I think it does exist because the term, […] you’ve maybe heard this; the heavy head. “He’s off with the heavy head. She’s off with the heavy head.” So there … so there probably is that … that stigma as well and there’ll be people probably in terms of personal pride, don’t want to go off sick and have as stress related illness.”*

***P22:** “Is a weakness. […] and it’s maybe just my perception, for talking’s sake, if I was to report absent tomorrow from duty … and if I was to cite, um, anxiety, depression … any … any form of … of emotional condition … or mental health condition, my … my … my perception would be, my career’s over … My career’s finished. . . . nobody wants a senior officer in charge of that … who’s had any form of mental health issues … ”*

***S3:** “Erm, and we have seen people, erm have very negative responses to disclosures of difficulty. Erm, and again, there has been that macho culture. And it’s more prevalent in some areas than others, actually, erm, but you see that kind of, well this is just the job, and you’ve just got to suck it up, and I’ve been dealing with that for years. And that complete lack of realisation that all our, all our reactions and levels of resilience are completely different. So just because you can handle it, doesn’t mean this person can. And actually, even though you think you’re handling it, it’s having an impact somewhere, it maybe just hasn’t happened yet.”*

Some maintained that the ‘male-dominated’ culture and representations of ‘masculinity’ prevent organisational MH interventions being successful and the negative experiences of officers that disclose personal difficulties is a deterrent to normalising and encouraging help-seeking for MH issues.

While several aspects of police cultures were deemed negative, others were considered positive and protective for wellbeing, including camaraderie, humour, and team dynamics that ‘galvanise’ people, cultivate a sense of belonging and serve a multitude of vocational purposes (see quotes below). This ‘sense of belonging’ was raised by both officers and stakeholders, but one stakeholder (S3) maintained that police staff (non-officers) do not experience the same team dynamics as police officers.

***S3:** “And there is a real sense of belonging to the police family. And I think, erm, people are, erm, very committed to helping one another. […] Um there’s probably something in that about, you know, taking up a role that’s a public servant type role. Erm, but they don’t just feel a duty to, to carry out their role for the public, they feel a duty for one another. So quite often, if somebody is struggling, erm, you will see the shift rally round, and try and provide support. Erm, so I think there is, there’s absolutely an opportunity through education to say, you know, this is an absolutely collective responsibility.”*

Current sources of support within the PSoS, including the *‘Your Wellbeing Matters’, ‘Your Time Matters’* and the Trauma Risk Management (TRiM) initiatives, among others, were deemed helpful and positive developments, but limitations to these approaches were also highlighted. *‘Your Wellbeing Matters’* aims to raise awareness of wellbeing issues and address the stigma around MH and included training 200 wellbeing champions, whose role is to signpost those who need support to relevant services. *‘Your Time Matters’* aims to record Superintendents’ working hours so that an informed case could be put forward to address the challenges of long-working hours. While this is obligatory, it is not taken up universally as officers said that ‘no-one checks it anyway’, and referred to it as ‘time consuming’ and ‘clunky’. The Trauma Risk Management (TRiM) process aims to identify risks and provide support for police staff and officers when they are directly involved in potentially traumatic incidents. Participants differed in their understanding of exactly what was involved in TRiM, but overall it was considered constructive and serving a good purpose. The extent to which police officers expressed they might seek support from their families varied; some suggested officers are now more likely to use family support to help prevent or address MH difficulties, while others preferred not to seek support from their family.

Efforts to address wellbeing and MH issues for police officers and staff were praised and acknowledged, but officers and stakeholders still felt more can be done, as stated by participant P19:

***P19:** “We’re, listen, we, we’re miles ahead of where we were before. But I’ll come back to my original point is, erm, we’re dealing with the symptoms, not the cause.”*

A variety of ideas about potential interventions to address MH and wellbeing issues were proposed. A common thread was the importance and agency of leadership, leading by example, and training to address stigma. Having ‘pioneers’, officers with experience of MH difficulties, coming forward and being open about their experiences, was suggested as a means of decreasing stigma, increasing dialogue and improving MH issues.

***S1 (Leadership):** “So I think definitely the superintendents need to lead on this and, and really ensure that their chief inspectors, inspectors and everyone down erm, are making this kind of almost normal, rather than, you know, abnormal for someone to put their hand up and say erm, “I’m not coping with this”, or whatever, you know. Or “I would like some … I would like to speak to someone, I need some help”, whatever. I think … yeah, I think erm, we need to get that message across so that no one feels like they don’t want … they’re embarrassed to do that in front of their colleagues … ”*

***P12 (Leadership):** “They need to remember who the workforce is. They keep on talking about … it’s a very old adage I was taught years ago here at the police college sergeant’s course, about inverting the pyramid. So the traditional pyramid of the chief is at the top and everybody’s … No, it’s not. The pyramid’s the other way around. The citizens are at the top, the officers and the staff who serve them and deliver a service to them are next. And then the sergeants, inspectors, chief inspectors. We as a management team should be supporting them and encouraging them and getting out of the way and removing the blockages to enable them to deliver a service. It doesn’t work that way. It works the other way around. So there is an absolute need for a change in complete mindset.”*

***P15 (Leadership):** “what the organisation does is create the start of an environment, it’s not a movement at that point in time, it’s just an environment. It’s … the people who then have the desire and occasionally the courage. […] There’s pioneers and there’s settlers. Um, and at the start of this, you need pioneers. Now, the unfortunate thing with pioneers is they’re at the front and they’re sticking their head above the parapet.”*

Another suggestion was a complete change in mindset in terms of the representations of the hierarchical workforce structure to improve wellbeing. Participants suggested several environmental changes as a pathway to healthier lifestyles and enhanced wellbeing, including better provision of workplace gyms and canteens, and policies around practical issues (e.g., limiting/prohibiting work-related smartphone use when off-duty, improving flexible working provision). They also highlighted a need for increased messaging and communication around MH issues.

***P10 (Policies & Environmental Changes):** “I think it’s looking at really practical things that change behaviour so big broad policy statements and initiatives and things like that are, erm, the culture is resilient to them, right. So you need to look at things that change behaviours. It’s the same way as, see if you smile a lot it makes you happy as opposed to when … when you’re happy you smile. So things like, it … it’s getting into the DNA of the organisation and say, well, what are the things that are causing that stress. So there’s a practical one for you straight away. […] Tell people that, unless you’re on call, your BlackBerry’s to be switched off.”*

***P6 (Policies & Environmental Changes):** “So sometimes it’s not the big, let’s have an initiative to get everybody out running at lunchtime or whatever. Simple things like, let’s get a canteen back in so actually we can sit for ten minutes. ‘Cause I think that’s probably better for your mental health, to have a … You know, I would have [to] actually leave my desk … for thirty minutes and go down and have a blether and a … chat and eat my food properly. Whereas mine’s is in the fridge and I’ll sit there by myself at my computer. So I actually think it would be healthier.”*

MH training – even mandatory training – was suggested as being beneficial. Mandatory counselling and health checks encompassing a mental, as well as physical, wellbeing focus were suggested to: reduce the stigma of consulting for MH issues; directly benefit individuals; make financial savings for the organisation (e.g., reducing sickness absence); and alleviate the pressures absences put on other staff.

***P14 (Training):** “And when we were sitting having our conversation about the [individual’s situation] and the way that […] the police don’t know how to deal with mental health, you don’t know how to deal with mental health. And I said, “No, you’re absolutely right, I don’t, because I’ve no training, I’ve had no experience really of various mental health”, you know, symptoms and what we can … you know … ”*

***P4 (Counselling & Health Checks):** “Yet, we’ve got an opportunity for our staff to go to wellbeing counselling, because their post is defined as being a risk, and they don’t take it up. […] but I think the organisation should make that mandatory. […] I find it ludicrous that I have officers who have got access to that service, and either, can’t find the time, or are discouraged from the, the attitude of their peers. So we, we’ve actually started writing it in their personal development conversation, that they’ve been offered, they’re encouraged to attend. But I would like to see the force make it mandatory. Because it’ll pick up other stuff, other than work, it’ll pick up home stuff […]”*

***P19 (Counselling & Health Checks):** “what we should be having is annual health checks. Annual health screening. So, not just for mental health, physical health, erm. So say, that health screening that, that I got, brilliant. […] And perhaps part of that should just be that general chat around about your overall wellbeing, what does that mean.”*

## Discussion

Our analysis of these interviews with PSoS Superintendents and stakeholders demonstrates a complex series of issues around the MH of police officers and staff. Participants discussed high levels of occupational stress and anxiety, either currently or in the past, consistent with previous studies (Arial et al., 2010; Chitra & Karunanidhi, 2018; Gershon et al., 2009; Goodman, 1990; Kroes, 1985; Kumarasamy et al., 2016; LaMontagne et al., 2016; Reiser, 1974). Underreporting of MH issues was considered widespread and experiences of perceived PTSD, anxiety and depression were reported. Participants who conveyed that they had experienced depression, connected their depression with occupational stressors. A lack of feeling valued played a notable role in depression; others made a direct link between operational trauma and perceived PTSD, even when symptoms did not overtly manifest themselves until triggered by circumstances long after an event. Stakeholders’ impression of how common PTSD is amongst officers appeared to diverge from that of the officers themselves, who tended to ‘play down’ PTSD experiences.

The main work-related stressors perceived to contribute to MH issues were job role, working hours/workload and organisational culture. Participants’ own experience, personal qualities and background impacted on their reactions to and experiences of operational stressors. Aligned with recent literature, organisational, as opposed to operational, stressors were reported by officers and staff to be the key stressors (Purba & Demou, 2019). The impact of significant organisational change (i.e. PSoS merged into a single police force in 2013) and the ways in which this change was applied still were seen to be the cause of a number of organisational stressors.

Stigma associated with MH in the workplace still prevents officers from being open about the challenges they are experiencing, although the stigma was not perceived to be as extreme as it once was. Bell and Eski (2016) reported a tendency for officers to be reluctant to seek support for MH issues for fear of being stigmatised, which could further exacerbate MH issues (Bell & Eski, 2016). Moreover, the masculine dynamics of police cultures and how these discourage officers from coming forward, as seen in previous research (Agocs et al., 2015; McCarty, 2013; McCarty et al., 2007), and being more open about MH difficulties, were also evident.

Overall, the results reflected a view that any future intervention strategy needs to acknowledge the importance of police cultures. This has been emphasized in literature which maintains that police identity and practice is constructed through police organisational culture (Tewksbury & Copenhaver, 2016). Such constructions ‘normalise’ emotional responses to the experiences and events police are likely to encounter and can have direct implications for any interventions that attempt to address stressors unique to police personnel (Carlier et al., 2000; Richardsen & Burke, 2007; Tewksbury & Copenhaver, 2016). Taking account of the broader organisation, culture is essential to a better understanding of the impact of changes within the organisation. Understanding the organisation and its structures is important to ensure interventions/intervention components can address existing barriers to help-seeking and engagement and capitalise on the opportunities to improve MH and wellbeing within the police. Going forward, calls were made for better provision of counselling, training and leadership and even suggestions for mandatory counselling. Police officers of all ranks need to be better informed about factors that can both provoke the development of or exacerbate mental health issues specifically within police organisations (Bell & Eski, 2015). Counselling provision for officers experiencing depression has been suggested previously, highlighting that such counselling should be made available without the potential for punitive implications of using such a service (Tewksbury & Copenhaver, 2016). Similar to previous research (Tewksbury & Copenhaver, 2016), our study participants were ambivalent about using counselling services as they found it difficult to be confident that doing so would not impede their career progression or trajectory. However, they maintained that a counselling programme could play a constructive role specifically through supporting attempts to find a better balance between one’s work and home life. Early screening, extra resources, workplace interventions and health promotion campaigns have been identified as potentially useful for improving adverse health-related behaviours and health issues (Penalba et al., 2008). Other intervention ideas to address mental health and wellbeing issues in the police workforce were suggested by participants. Early recognition of PTSD symptoms, for example, is essential in making the diagnosis of post-traumatic stress in high-risk occupational cohorts such as police forces (Austin-Ketch et al., 2012). The importance of training to address and effectively manage MH issues was a common thread. This included training aimed at line managers, to provide them with the tools to identify and manage MH difficulties and minimize exclusion within the workplace. Other studies have also suggested that if knowledge is improved upon, the detrimental impact of mental health difficulties can be circumvented for the police workforce and possibly even for the communities that the police organisation serves (Bell & Eski, 2015; Mitchell et al., 2001). Participants also spoke about the importance of leadership and in particular, leading by example with regards to mental health issues in the police force. One participant maintained that what is needed is individuals across ranks to be more open about the mental health difficulties they are experiencing, despite the vulnerable position this would put them in. Encouraging officers and their spouses to enter confidential counselling, altering training and hiring practices, making peer counsellors available, establishing administrative changes, adding diversity programs and critical incident training with the aim to minimize the risk of work stress among police officers are other suggestions reported in the literature (5).

Our study has a number of strengths and limitations. As the topic of MH remains sensitive, a key strength of this research is that independent university researchers conducted the participant interviews and analysis, providing an added layer of privacy protection and strong reassurance of participant confidentiality. Overall, participants were keen to provide their perspectives on the MH issues which they perceived to be prevalent or problematic within their workforce. This is despite participants recognising a widespread fear that such information could be ‘used against them’ if there was any risk that their views and experiences were expressed to peers and line managers.

Having the PSoS Superintendents as the target group for our sample, allowed us to gain insights into how mental health issues may manifest themselves in a group that are exposed to a large number of traumatic incidents, whose role places high levels on demand on them, who have come through the ranks and are likely to also be involved in line-managing more junior staff. While we cannot be certain of the generalisability of our results across the PSoS, recruiting almost a fifth of all PSoS Superintendetns (30 Superintendents; 2:1 male: female ratio) ensured we have a wide coverage of experiences and perceptions across this rank. It is also important to acknowledge limitations. Thus, only sampling within the Superintendent ranks, a decision which was in part determined by the resources available for the study, could be seen as a limitation, as we are not capturing current personal experiences of officers of other ranks, only those recalled by the superintendents when they were at earlier stages of their career. Inevitably, this may not provide an accurate representation of all of the mental health challenges experienced currently by more junior ranks, either because of issues related to retrospective recall or due to the changing nature of mental health stressors. A further limitation is that the number that we were able to interview within the Superintendent rank has not allowed us to examine perceived differences in gender, ethnicity, job title and geography, because of potential risks to (presumed) deductive disclosure where there are small numbers of people with particular combinations of these characteristics within PSoS. Thus, as the data we gathered are very rich, often describing very personal experiences, subgroup analysis could potentially compromise participants’ anonymity.

## Conclusions

This partnership project was initiated by Police Scotland, as part of their ongoing work to promote and manage the health and wellbeing of their. Recognition of the importance of good MH and the challenges associated with raising MH issues in police workforces (and indeed many other occupational groups) highlighted the need for a study to assess and understand MH needs within Police Scotland; and identify new ideas for interventions that would be acceptable for better management of MH issues. In line with studies across other police forces, the main perceived stressors reported by our participants were: long working hours, workload, culture, leadership and organisational change. Officers and staff recognised that there had been progress towards promoting and managing MH in the service but identified further potential for interventions, including training, counselling, and environmental workplace changes which could further address continuing mental health issues within police cultures. Whilst results from published studies report some positive results, pointing to the potential effectiveness of some interventions, they also highlight the need for more research to understand the interventions and/or intervention components that are required for the prevention and management of MH issues in this workforce. It is essential that these are feasible to implement, acceptable for the workforce and the organisation, and effective and cost-effective in improving mental health in police officers and staff (Penalba et al., 2008). Future larger studies with police officers and staff across forces, and particularly studies which can examine perceived differences in mental health issues by gender, age, rank, and ethnicity, are warranted to better understand the needs of the changing workforce. Our findings can serve as the groundwork to inform workplace interventions for promoting good MH and managing mental illness in the workplace, tailored to the needs of PSoS and its workforce while taking into consideration organisational specific opportunities and barriers.
